# Adduct under Field—A Qualitative Approach to Account for Solvent Effect on Hydrogen Bonding

**DOI:** 10.3390/molecules25030436

**Published:** 2020-01-21

**Authors:** Ilya G. Shenderovich, Gleb S. Denisov

**Affiliations:** 1 Institute of Organic Chemistry, University of Regensburg, Universitaetstrasse 31, 93053 Regensburg, Germany; 2 Department of Physics, Saint-Petersburg State University, 198504 Saint-Petersburg, Russia; gldenisov@yandex.ru

**Keywords:** solvent effect, hydrogen bond, NMR, condensed matter, polarizable continuum model, reaction field, external electric field, proton transfer

## Abstract

The location of a mobile proton in acid-base complexes in aprotic solvents can be predicted using a simplified Adduct under Field (AuF) approach, where solute–solvent effects on the geometry of hydrogen bond are simulated using a fictitious external electric field. The parameters of the field have been estimated using experimental data on acid-base complexes in CDF_3_/CDClF_2_. With some limitations, they can be applied to the chemically similar CHCl_3_ and CH_2_Cl_2_. The obtained data indicate that the solute–solvent effects are critically important regardless of the type of complexes. The temperature dependences of the strength and fluctuation rate of the field explain the behavior of experimentally measured parameters.

## 1. Introduction

Proton transfer represents the simplest possible chemical reaction [1] and is ubiquitous in chemistry [2,3], material science [4,5,6], and biology [7,8]. In the latter case, the complexity of the process can increase to a hydrogen atom transfer [9,10]. In condensed matter, the mechanism and the pathway of proton transfer depend on the local environment. As a result, the study of proton transfer processes in a given system can be used as a tool to study the local environment. In most cases, it will require a theoretical simulation of the proton transfer under question. Such simulations are still very challenging as they depend on a compromise between the size of the modeled molecular system and the quality of accounting for intermolecular interactions. The size should be large enough to include all relevant interactions; the quality should be good enough to estimate their effect correctly. One may prefer to simulate a given molecular system in condensed matter using oversimplified approaches, looking only for a qualitative description of the system. Often, such approaches are fully justified. The available theories of nonadiabatic [11] and adiabatic [12] proton transfer reactions provide a useful background for understanding experimental results as on reversible proton transfer in the Zundel cation [13,14,15] as well as on fast proton dynamics in general [16,17,18]. The precision of such analysis can be improved further [19]. However, the most challenging part is to account for the effect of fluctuating solute–solvent interactions [20,21,22].

Often, one needs to restrict proton mobility in order to stabilize individual structures. This is especially important for high-resolution nuclear magnetic resonance spectroscopy (NMR) whose characteristic time is of the order of 10^−3^ s. Basically, proton and molecular exchange can be suppressed by lowering the temperature. However, when studying intermolecular interactions in solution, one is strictly limited with the available temperature range. For aprotic polar solvents, the lowest possible temperature is about 100 K [23]. This temperature is not always low enough to affect proton dynamics [24,25]. Another problem is that the required temperature depends on intermolecular interactions in a complex way [26,27,28]. Even the structures of complexes with strong noncovalent interactions are affected by interactions with the environment [29,30,31,32]. In solution, this can be visualized by molecular dynamics simulations [33,34]. Thus, conventional gas-phase calculations can neither be used to predict at what temperature in a given molecular system proton exchange can be suppressed in a given solvent nor to simulate the mean structure of the system in solution.

Solvent effect can be divided into two parts: (i) fluctuating solute–solvent specific interactions and (ii) macroscopic electric field. The polarizable continuum model (PCM) includes only the latter effect [35]. As a result, this model is not sufficient to simulate the structure of noncovalently bound complexes in polar solvents [36]. The effects of specific solute–solvent interactions are to some extent implicitly included in the SMD (Solvation Model based on Density) model [37]. This model uses a number of solvent-specific parameters. In reality, the tabulated values of Abraham’s hydrogen bond acidity and basicity, aromaticity, and electronegative halogenicity of solvents are not always the optimal choice for a given molecular system. The temperature dependence of these parameters is not known. Thus, given standard conditions, this model can be a good approximation and would fail otherwise. The problem can be overcome by using molecular dynamics approaches. However, they are computationally consuming and challenging when in non-aqueous solutions [38].

**Figure 1:**
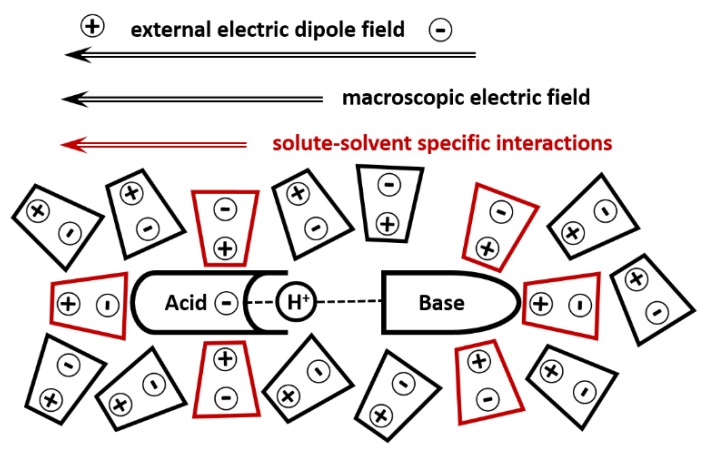
The direction of the external electric field that simulates the effect of solute–solvent interactions on the H-bond.

Alternatively, the effect of environment can be simulated using a fictitious external electric field [39,40,41]. The main advantage of this approach in relation to complexes with noncovalent interactions is that their experimental structures can be reproduced using only one parameter—the strength of the external electric field. The physical meaning of this field is illustrated in Figure 1. For the sake of simplicity, we consider a hydrogen-bonded (H-bond) complex in an aprotic polar solvent. The strongest intermolecular interaction in this complex is the acid-base H-bond. The geometry of this bond is affected by the macroscopic electric field generated by dipole moments of solvent molecules. This effect can be simulated by the PCM model. Besides that, there are weak yet multiple interactions with solvent molecules. They also cause changes in the electron density in the acid and base that affect the position of the mobile proton. The PCM model ignores this effect. The SMD model can include this effect through the empirical parameters. Using the external electric field model, we can estimate the relative amplitudes of both macroscopic and specific effects on the properties of the hydrogen bond under question when simulating a given experimental property of H-bond with the external field alone and in combination with the PCM model. The efficiency of this Adduct under Field (AuF) approach was demonstrated using a complex of hydrogen fluoride with pyridine [42].

**Figure 2:**
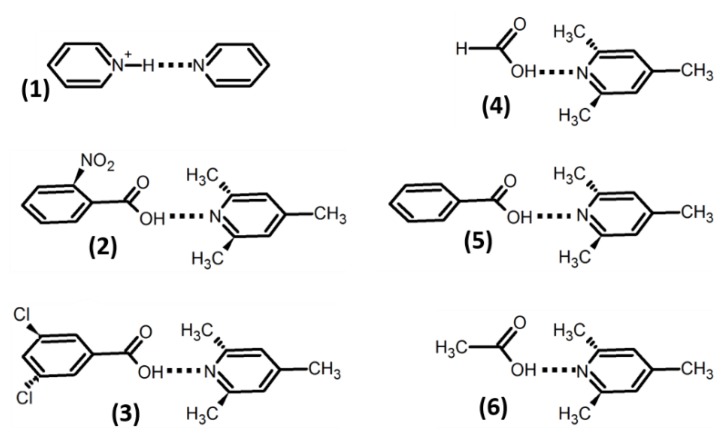
H-bonded complexes studied in this paper: proton-bound homodimer of pyridine (**1**) and complexes of 2,4,6-trimethylpyridine (collidine) with 2-nitrobenzoic acid (**2**), 3,5-dichlorobenzoic acid (**3**), formic acid (**4**), benzoic acid (**5**), and acetic acid (**6**).

In this work, we use the AuF model in order to simulate experimentally observed solvent-driven proton transfer in a number of H-bonded complexes. The aim of this study is to formulate a simplified computational approach capable of predicting the temperature at which proton exchange will be suppressed in any given solute–solvent system. The model molecular systems are shown in Figure 2. These complexes have been experimentally studied in the past in a liquid CDF_3_/CDClF_2_ mixture, exhibiting a dielectric constant between 20 at 170 K and 38 at 103 K [23]. The proton-bound homodimer of pyridine (**1**) does not have chemically active sites exposed to the solvent while the carboxylic moiety in **2**–**6** can specifically interact with solvent molecules [34].

Proton-bound homodimers can be of two types—symmetric, in which case the partners equally share the binding proton, and asymmetric, where the proton has a stronger bond to one of the partners at any given moment in time [43]. In the proton-bound homodimers of pyridine derivatives in CDF_3_/CDClF_2_ mixtures, the mobile proton jumps between the two bases faster than 10^3^ s^−1^ down to 120 K [44] and slower than 10^11^ s^−1^ up to 290 K [45].

In Table 1 ^1^*J*(^15^N^1^H) scalar coupling constants in **2**–**6** in CDF_3_/CDClF_2_ solution are collected [46]. These constants were measured at different temperatures—the reason being that above these temperatures the solvent-driven exchange between O-H···N and O^−^···[H-N]^+^ forms of the complexes was fast on the NMR time scale. Proton tautomerism in such complexes has previously been studied in detail [34]. For our purpose, it is important that the process strongly depends on the p*K*_a_ of the involved acid. As a result, the solute–solvent interactions can be analyzed in a large temperature range. We know that in the O^−^···[H-N]^+^ form ^1^*J*(^15^N^1^H) ≳ 90 Hz [47]. Thus, for some of these complexes, the tautomerism can be slow on the NMR time scale of chemical shifts and fast on the NMR time scale of scalar couplings. However, such aspects are beyond the precision of our qualitative model.

## 2. Results

### 2.1. Proton-Bound Homodimer of Pyridine

**Figure 3:**
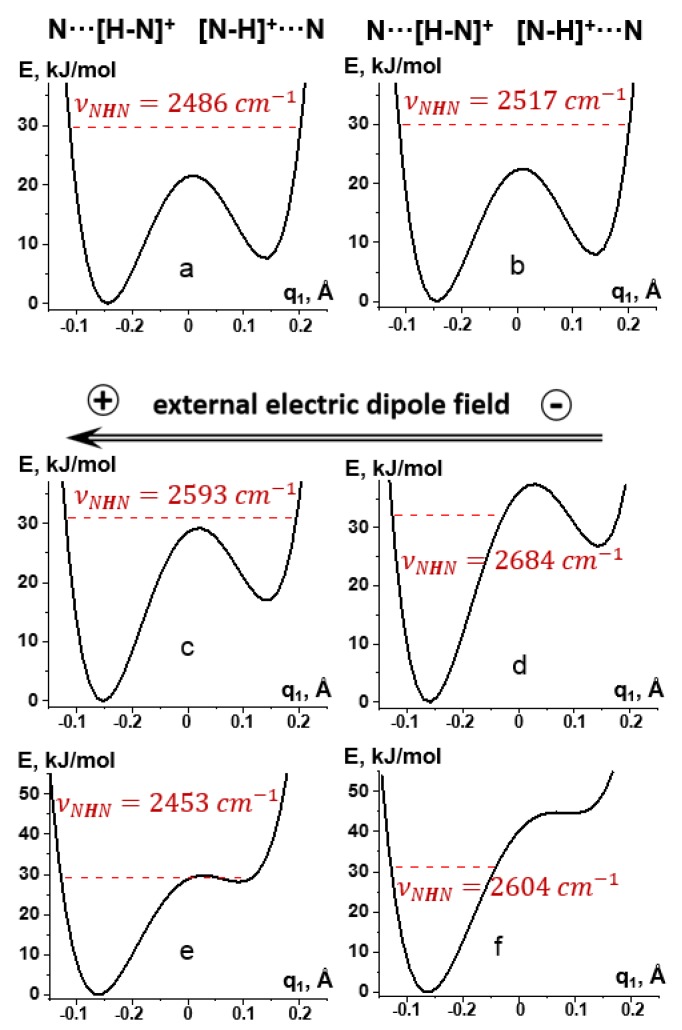
Potential energy curve of a proton transfer within **1** at different approximations: (**a**) PCM (ε=29.3), (**b**) SMD (CH_2_Cl_2_, ε=8.9), (**c**) PCM (ε=29.3) and the external electric field of 0.001 a.u., (**d**) PCM (ε=29.3) and the external electric field of 0.002 a.u., (**e**) the external electric field of 0.003 a.u., and (**f**) the external electric field of 0.005 a.u. Dashed lines indicate the energy of the ground state. $\nu_{NHN}$ are the frequencies of the mobile proton stretching vibration. q_1_ corresponds to the distance of the mobile proton with respect to the H-bond center [51].

Figure 3a shows the potential energy curve of a non-adiabatic proton transfer in **1** under the PCM approximation at ε = 29.3. The minimum energy structures of the pyridines of **1** are not equal. Therefore, when the mobile proton is transferred from one pyridine to the other while all other atoms are fixed, the second minimum has a larger energy. In reality, this fictitious profile is not present and only shown to illustrate further changes. The ground vibrational level of the proton is higher than the energy of the transition state. The frequency of the stretching vibration ($\nu_{NHN}$) estimated under the harmonic approximation is 2486 cm^−1^. While the potential is anharmonic, this value is a rough estimate and is given for illustrative purposes only [48]. The accuracy of the calculations can only be increased at the cost of making them very time-consuming [49,50]. The value of ε can also be challenged; in CDF_3_/CDClF_2_ solution at about 130 K ε ≈ 30 [23]. However, the non-adiabatic proton transfer depends on an optical dielectric constant of about 2 [16]. Under the gas phase harmonic approximation, $\nu_{NHN}$ = 2142 cm^−1^. Thus, a qualitatively similar potential surface will be observed for any value of ε. We are interested in the situation when this transfer is suppressed. What is important is that solvent polarization alone cannot cause this effect.

CDF_3_/CDClF_2_ solution cannot be simulated using the SMD approximation because its parameters are not known. Instead, chemically similar CH_2_Cl_2_ can be used. Although $\nu_{NHN}$ increases under this approximation to 2517 cm^−1^, it is still higher than the energy of the transition state, Figure 3b—meaning that this model cannot reproduce the experimentally observed single-well location of the mobile proton in **1**.

The single-well location becomes possible in the presence of an external electric field. Under the PCM approximation (ε = 29.3) and the field of 0.001 a.u., the energy of the ground vibrational level of the mobile proton is very close to the energy of the transition state, Figure 3c. At 0.002 a.u., the former is lower than the latter, Figure 3d. This increase of the field is accompanied by an increase of $\nu_{NHN}$ from 2593 cm^−1^ to 2684 cm^−1^, Figure 3c,d. Thus, the experimentally observed proton jumps in **1** can be simulated under the PCM approximation and ε ≈ 30 when the strength of the external field is above 0.001–0.002 a.u. There is no hard criteria for choosing the most appropriate value of the field. We can only state that the lower limit of its strength is 0.001 a.u. Within the gas phase approximation, this limits increases to at least 0.003 a.u., Figure 3e,f.

### 2.2. Complexes of Collidine with Acids

In Table 2, geometric parameters of H-bond in **4** under different approximations are reported. Although these parameters depend on the level of approximation, the mobile proton is located at the acid in all cases. Only at ε > 29 do there appear higher energy local minima on the potential energy curve of proton transfer that correspond to the proton location at collidine. Taking into account a qualitative character of our analysis, we studied the effect of the external electric field on the location of the mobile proton in **2**–**6** at a computationally efficient *w*B97XD/def2svp approximation. We also restricted our analysis to the comparison of the difference between the energies of the two minima (proton at acid and proton at base) on the potential energy curve of proton transfer. The values of ε under the PCM approximation were taken equal to 12.5 for **2** and 29.3 for **3**–**6**. These values are close to the dielectric constant of CDF_3_/CDClF_2_ solution at 200 K and 130 K, respectively [23]. There is no need to select ε with a higher precision because Table 2 clearly demonstrates that, at ε > 10, its effect on H-bond geometry remains rather constant.

**Figure 4:**
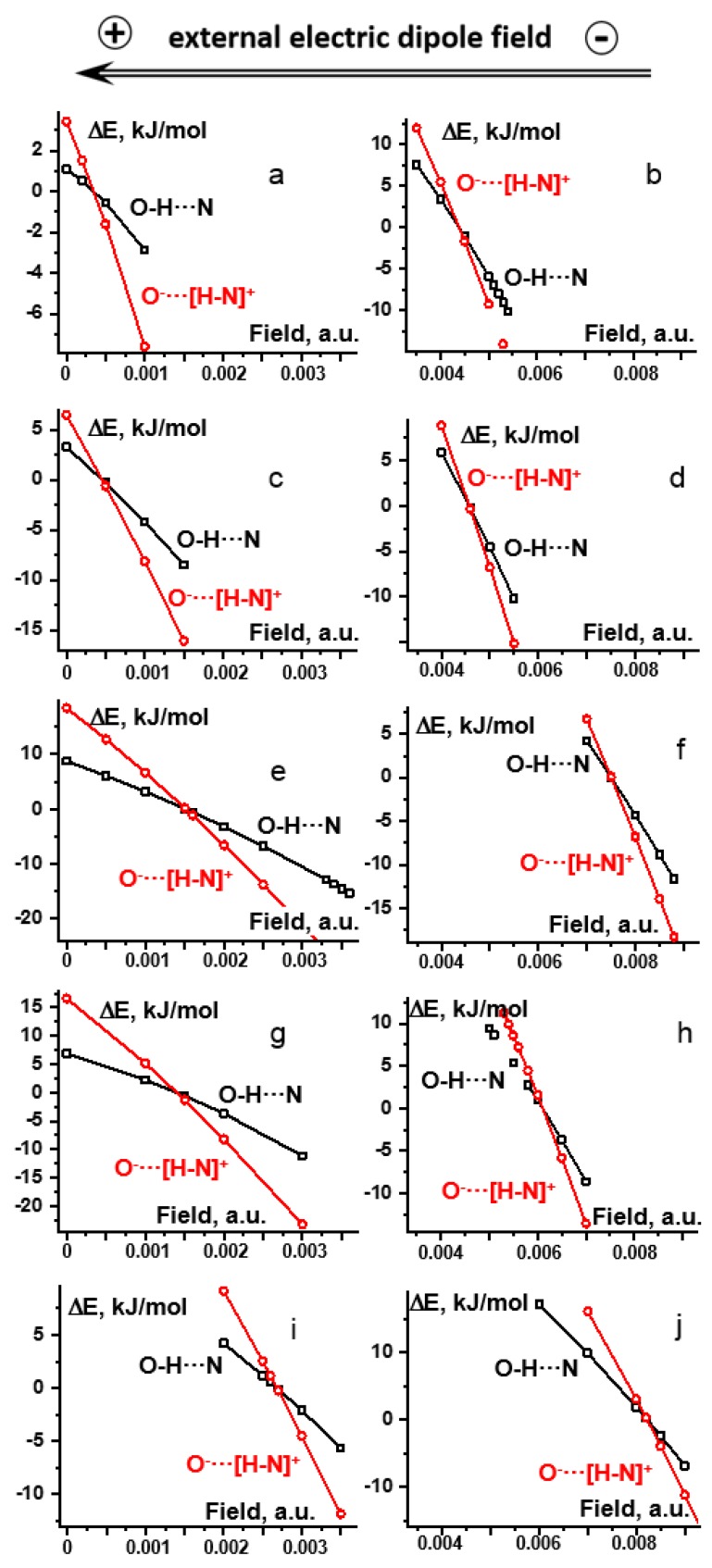
The effect of the external electric field on the energy of O-H···N (black □) and O^−^···[H-N]^+^ (red ○) forms of H-bonds in **2**–**6**. For each complex, there is a unique value of the field strength for which the energy minima of the two forms are equal. ΔE corresponds to the energy with respect to the value at this field. **2**: (**a**) PCM (ε=12.5), (**b**) gas-phase; **3**: (**c**) PCM (ε=29.3), (**d**) gas-phase; **4**: (**e**) PCM (ε=29.3), (**f**) gas-phase; **5**: (**g**) PCM (ε=29.3), (**h**) gas-phase; **6**: (**i**) PCM (ε=29.3), (**j**) gas-phase.

Figure 4 demonstrates the effect of the external electric field on the energy of the molecular (O-H···N) and ionic (O^−^···[H-N]^+^) forms of H-bonds in **2** (Figure 4a,b), **3** (Figure 4c,d), **4** (Figure 4e,f), **5** (Figure 4g,h), and **6** (Figure 4i,j) under the PCM and gas-phase approximations. For all complexes in both approximations, an increase of the field causes an energy decrease of both forms, although the favor is towards the ionic one. Upon this increase, the profile of a potential energy curve changes from a single-well (molecular) to a double-well to a single-well (ionic) one. The double-well potential interval is shown in Figure 4. For each complex, there is a unique value of the field strength for which the energy minima of the two forms are equal. ΔE corresponds to the energy of the complex with respect to the value at this field.

Strictly speaking, in order to find which value of the external field is the best approximation of experimental conditions, one needs (i) to estimate the molar fractions of the two forms from NMR spectra and (ii) to then find at what field the same ratio is be obtained in calculations. The former can be done using either the value of ^1^*J*(^15^N^1^H) in Table 1 or the ^1^H-NMR chemical shift of the mobile proton [52]; the latter—by calculating the effect of the field on the free energy. However, both of these estimates are rough and are redundant in the case of the present qualitative analysis. Instead, the lower limit of the external electric field can be associated to the value at which the energy minima of the two forms are equal, Table 3.

### 2.3. The Gas-Phase Proton Affinities

For the further discussion of the obtained results, we will use the values of the gas-phase proton affinities (PA). These values are listed in Table 4 for a number of selected proton acceptors.

## 3. Discussion

**Figure 5:**
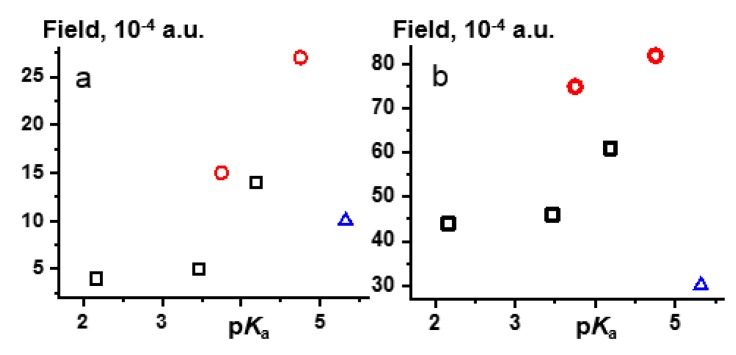
The lower limit of the external electric field simulated the effect of CDF_3_/CDClF_2_ on **2**, **3**, **5** (black □), **4**, **6** (red ○), and **1** (blue Δ) under the PCM (**a**) and gas-phase (**b**) approximations as a function of the p*K*_a_ of the proton-donor.

Figure 5 shows lower limits of the external electric fields simulated the effect of CDF_3_/CDClF_2_ on **1**-**6** under the PCM (5a) and gas-phase (5b) approximations as a function of the p*K*_a_ of the proton-donor. The p*K*_a_ of pyridine is 5.32 [46]; other values are listed in Table 1; Table 3. For **2**–**6**, the strength of the field required to transfer the proton to the base correlates with the strength of the acids in both approaches. **1** deviates strongly from these correlations as it should. The energy minima of two tautomeric forms of **1** are equal at zero field. The values shown for **1** (Figure 5) correspond to the case when this double-well potential energy curve becomes a single-well one (Figure 3). However, in contrast to **2**–**6**, proton tautomerism in **1** remains fast on the NMR time scale. Thus, the physical meanings of the values reported here for **2**–**6** and **1** are different. What is important is that (i) the order of magnitude of the electric field simulated the effect of CDF_3_/CDClF_2_ on **1**, **2**–**6**, and pyridine⋯HF⋯(HCF_3_)_n_ [42] is the same and (ii) its effect on H-bond geometry correlates with the proton donating power of involved acids. The former means that the AuF approach is appropriate for a qualitative description of solute–solvent interactions. The latter suggests that it should be possible to predict the effect of a given solvent on the geometry of a given H-bonded complex. What is the most reliable representation of the correlation between the expected strength of the external electric field and the chemical properties of involved acids and bases?

The use of p*K*_a_ as a measure of acid’s proton-donating power in non-aqueous solutions introduces an error into the correlation. The reason is that the p*K*_a_ depends on solvation in water that is very specific solvent [53,54,55]. The pKa‘s of ionizable groups in a non-aqueous environment can be estimated theoretically [56]. However, such calculations are quite demanding. Alternatively, they can be empirically corrected to a solvent under question [57]. The easiest way to estimate the proton-donating and proton-accepting powers is to calculate the gas-phase proton affinity (PA), Table 4 [58,59]. These values are very close to available experimental data for pyridine (930 kJ/mol) [60,61], collidine (980 kJ/mol) [61], benzoate (1422 kJ/mol) and 2-nitrobenzoate (1383 kJ/mol) [62], formate (1445 kJ/mol) and acetate (1456 kJ/mol) [63], and fluoride (1550 kJ/mol) [64]. 

**Figure 6:**
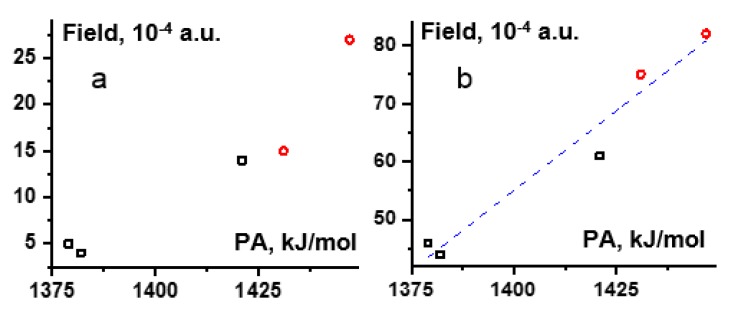
The lower limit of the external electric field simulated the effect of CDF_3_/CDClF_2_ on **2**, **3**, **5** (black □) and **4**, **6** (red ○) under the PCM (**a**) and gas-phase (**b**) approximations as a function of the PA of the involved acids.

Figure 6 demonstrates the lower limits of the external electric field simulated the effect of CDF_3_/CDClF_2_ on **2**–**6** under the PCM (Figure 6a) and gas-phase (Figure 6b) approximations as a function of the PA of the involved conjugate bases. We are aware that the use of the PCM approximation perturbs such correlations due to its dependence on the size of a molecular complex under study [58]. Therefore, we intend to use the gas-phase approximation. The analytical expression for the correlation shown in Figure 6b is:

$$F^{gas}\left[in\;a.u.\right]=\left\{\left(0.55\pm 0.07\right)\cdot PA\left[in\;kJ/mol\right]-\left(700\pm 100\right)\right\}\cdot 10^{-4}.$$

**Figure 7:**
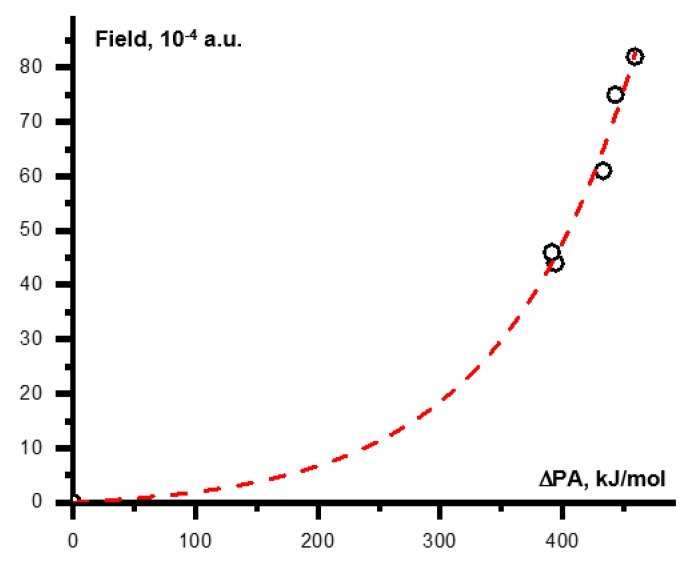
A functional dependence between the lower limit of the external electric field at which the energy minima of the Acid-H···Base and Acid^−^···[H-Base]^+^ forms of an H-bonded complex in CDF_3_/CDClF_2_ are equal and the difference between the PAs of a proton donor and an acceptor, ΔPA.

This correlation can be generalized by replacing the PA of the conjugate bases with a difference between the PA’s of a proton donor (conjugate base) and an acceptor: $\Delta PA=PA^{donor}-PA^{acceptor}$. The analytical expression for this final correlation as shown in Figure 7 is:

$$F^{gas}\left[in\;a.u.\right]=1.3\cdot 10^{-4}\cdot \left\{\exp\left(0.009\cdot \Delta PA\left[in\;kJ/mol\right]\right)-1\right\}.$$

Here, $F^{gas}$ tends to zero as ΔPA tends to zero that is physically correct. Results obtained for **1**, **2**, and **6** provide limiting values for the strengths of the external electric field simulated the effect of CDF_3_/CDClF_2_ on H-bond geometry at 300 K, 200 K, and 100 K, respectively. When the gas-phase approximation is used for the field-strength calculations, these values are about 0.003 a.u., 0.004 a.u., and 0.082 a.u., respectively. Only a part of this field can be associated to solvent polarization and accounted for in the frameworks of the PCM approach. The effect of this contribution on H-bond geometry is roughly constant and temperature independent. Another part of the field simulates the effect of solute–solvent interactions. Their impact is fluctuating and depends on temperature. Let us estimate the magnitudes of these two contributions.

For **1**, the lower limit of the external electric field estimated under the PCM approximations is about 0.001 a.u. This value can be associated to the effect of solute–solvent interactions. Notice that both pyridines of **1** are affected by these interactions—meaning that 0.001 a.u. reflects the difference between the effects of solvation on the protonated pyridine and the H-bonded one. This value fluctuates faster than 10^3^ s^−1^ down to 120 K and slower than 10^11^ s^−1^ up to 290 K [44,45]. As a result, proton exchange within **1** is fast on the NMR and slow on the IR time scales in this temperature range.

Proton tautomerism *acceptor···H-donor* ⇌ *[acceptor-H]^+^···(donor)^-^* in CDF_3_/CDClF_2_ is strongly shifted towards the *[acceptor-H]^+^···(donor)^-^* tautomer already at 200 K when the difference between the PAs of the proton donor and the acceptor is smaller than 400 kJ/mol, **2**. The larger the difference, the lower the temperature should be. In CDF_3_/CDClF_2_, at the lowest experimentally achievable temperature of 100 K, this tautomer dominates completely only when ΔPA is smaller than 500 kJ/mol, **6**. The lower limits of the external electric field estimated under the PCM approximations required to stabilize the *[acceptor-H]^+^···(donor)^-^* tautomers of **2**–**6** in CDF_3_/CDClF_2_ vary from 0.0004 a.u. to 0.0027 a.u., Table 3. However, in contrast to **1,** these values fluctuate slow on the NMR time scale. Tentatively, this field can mostly be associated to solvation of the carbonyl group. The lower the temperature, the more stable the interaction with solvent molecules. For a rough estimate, it can be assumed that the value of this slow fluctuating field increases from 0.0005 a.u. to 0.0030 a.u. in the temperature range from 200 K to 100 K.

For the chemically similar CH_2_Cl_2_, the lowest experimentally achievable temperature is about 170 K [27]. Thus, when ΔPA is smaller than 400 kJ/mol, the *[acceptor-H]^+^···(donor)^-^* tautomer should dominate. This estimate can be checked using a complex of 4-nitrophenol with acetate in CD_2_Cl_2_ [65]. In this complex, $\Delta PA=PA^{acetate}-PA^{phenolate}=93\;kJ/mol,$ Table 4. At 173 K, the *phenol···(acetate)^-^* form of the complex dominated. Ab initio molecular dynamics demonstrated that this form was stabilized by interactions of the carbonyl group with solvent molecules. This interaction is implicitly included in our correlation. Formally speaking, these results support our estimate. However, molecular dynamics showed that the location of a tetraalkylammonium anion was also critically important in this case. This interaction is not covered by our correlation. This effect is absent for charged H-bonded complexes only for very bulky anions [25]. Notice that solvation of the phenolate oxygen will reduce the effect of the carbonyl solvation.

The importance of specific interactions is extreme in the case of a complex of pyridine with hydrogen fluoride. This complex was studied by NMR [23,66] and model calculations [43,67]. The strength of the external electric field, at which the experimental geometry of the N⋯H and H-F bonds of pyridine⋯HF in CDF_3_/CDClF_2_ is reproduced, depends on the number of the solvent molecules coordinated to the fluorine in model adducts pyridine⋯HF⋯(HCF_3_)_n_. It is about 0.017 a.u. for pyridine⋯HF, 0.010 a.u. for pyridine⋯HF⋯HCF_3_, and 0.006 a.u. for pyridine⋯HF⋯(HCF_3_)_2_. For the former adduct, ΔPA=611 kJ/mol, Table 4. For pyridine⋯HF⋯HCF_3_, the structure of the proton donor is HF⋯HCF_3_ and ΔPA= 493 kJ/mol. It is not clear how to estimate the PA of F^-^⋯(HCF_3_)_2_ because the structure of such composite donor critically depends on its protonation state. In any case, it should be smaller than 500 kJ/mol that explains a near central location of the mobile proton between the nitrogen and fluorine atoms as observed in experiments. Thus, also for this complex, our qualitative analysis agrees with a high-level molecular dynamics [33].

## 4. Materials and Methods 

Gaussian 09.D.01 program package was used [68]. If not stated otherwise, geometry optimizations were done at the *w*B97XD/def2tzvpp and *w*B97XD/def2svp approximations for **1** and **2**–**6**, respectively [69,70]. The identity of minima was confirmed by the absence of imaginary vibrational frequencies. The default SCRF=PCM method has been used to construct the solute cavity. The parameters for SMD calculations were adapted from the Minnesota Solvent Descriptor Database: eps = 8.93, epsinf = 1.4242, H-bond acidity = 0.1, H-bond basicity = 0.05, surface tension at interface = 39.15, carbon aromaticity = 0.0, electronegative halogenicity = 0.667 [71]. Although the SMD model was parametrized for the Minnesota functionals family, for the qualitative analysis presented in this work, we decided to use the same functional for all types of calculations. 

The external electric field was added to calculations using a keyword Field. The C_2_ symmetry axis of pyridine or collidine was fixed along the direction of the field using a keyword Z-Matrix. The electric dipole field in Gaussian is directed from the negative to the positive potential that is opposite to the conventional direction of electric field.

The gas-phase proton affinities (PA) were calculated as follows:

$$PA=\Delta H^{298}\left(B\right)+5RT/2-\Delta H^{298}\left(BH\right).$$

Here, $\Delta H^{298}\left(B\right)$ and $\Delta H^{298}\left(BH\right)$ stand for the sums of the electronic and thermal enthalpies of a base and its conjugate acid or the conjugate base of an acid and the acid at 298 K. The enthalpies were estimated at the B3LYP/6-311++g(3df,2p) level. This level provides a reasonable description of the structure and harmonic frequencies of the neutral and charged H-bonded systems in the gas phase [72]. It is also sufficient to obtain correct values of enthalpies [73].

## 5. Conclusions

The gas-phase proton affinity (PA) of conjugate bases is larger than that of most neutral bases. Proton transfer in condensed matter requires either an H-bond network [74] or solvation [54,75]. In specific cases, small alterations can cause pronounced changes [76]. Therefore, neither gas-phase nor PCM calculations can reproduce the geometry of an acid-base complex in condensed matter, if its environment is ignored. In contrast, useful qualitative data can be obtained using the Adduct under Field (AuF) approach. The weak yet multiple interactions between the acid-base complex and solvent molecules influence the electron density in the acid and base that affects the position of the mobile proton. These changes can be simulated using a fictitious external electric field. The macroscopic electric field can be either accounted for by the PCM approach or included in the strength of the field. The strength of the solute–solvent interactions fluctuates and its effective magnitude depends on temperature. In this paper, we report estimates of the strength of the fictitious field that simulates solvation effect of CDF_3_/CDClF_2_ on homo- and heterogeneous acid-base complexes in the temperature range from 300 K to 100 K. With some limitations, the obtained results can be extended onto the chemically similar CHCl_3_ and CH_2_Cl_2_. The computational simplicity of the AuF approach could lend itself to wide application including large molecular systems [77,78,79,80].

In the presence of the external electric field, the potential energy curve of a proton transfer within the proton-bound homodimers of pyridines changes from a symmetric double-well potential to an asymmetric single-well one. In the temperature range 120–290 K, the fluctuation rate of this field is between 10^3^ and 10^11^ s^−1^ that defines the rate of proton exchange within the homodimers. The lower limits of this field are reported above. For [FHF]^−^ [81,82] or [H_2n+1_O_n_]^+^ [83] proton-bound homodimers, the same strength of the field can be an acceptable approximation only when several solvent molecules are explicitly included into calculations.

Below 200 K, solvent effects on heterogeneous acid-base complexes can be simulated using a quasi-constant fictitious field. For complexes of pyridine with carboxylic acids, the strength of this field and its temperature dependence are reported above. For complexes of pyridine with alcohols and phenols, the strength will be smaller because interaction of the carbonyl oxygen with solvent molecules increases the proton-donating power of the hydroxylic group of carboxylic acids. When a proton-donating or proton-accepting center is open for a strong interaction with solvent molecules, these molecules should be included into the model adduct. See, for example, pyridine⋯HF⋯HCF_3_ and pyridine⋯HF⋯(HCF_3_)_2_ adducts.

The most important conclusion of this study is that solute–solvent interactions remarkably affect the geometry of acid-base complexes in aprotic solvents even if the active sites of these complexes are not accessible for solvent molecules. As a result, these complexes exhibit proton tautomerism *acceptor···H-donor* ⇌ *[acceptor-H]^+^···(donor)^−^* in a large temperature range. The rate of this process is often slow on the time scales of electronic excitations and molecular vibrations while fast on the time scale of NMR. Therefore, both tautomers can be observed in the former cases while exchange averaged parameters will be obtained in the latter. Only in the presence of moderately strong solvation effects, for example, when solvent molecules interact with the proton-donating group, can the lifetime of the *[acceptor-H]^+^···(donor)^−^* tautomer become long on the NMR time scale in the temperature range from 200 K to 100 K.

## Figures and Tables

**Table 1:** Experimental ^1^*J*(^15^N^1^H) scalar couplings in **2**–**6** in CDF_3_/CDClF_2_ solution [46].

Complex	^1^*J*(^15^N^1^H), Hz	T, K	p*K*_a_^1^
**2**	−87.0	200	2.16
**3**	−81.1	130	3.46
**4**	−79.1	120	3.75
**5**	−76.9	120	4.19
**6**	−65.4	110	4.75

^1^ The p*K*_a_’s of the involved acids.

**Table 2:** H-bond geometry of **4** under different DFT (Density Functional Theory) approximations.

DFT Functional	Basis Set	PCM, ε	N···H, Å	N…O, Å
wB97XD	def2svp	−	1.689	2.696
wB97XD	def2svpp	−	1.655	2.674
wB97XD	def2tzvp	−	1.701	2.709
wB97XD	def2tzvpp	−	1.707	2.712
B2PLYPD3, gd3	def2svp	−	1.692	2.697
B2PLYPD3, gd3	def2svpp	−	1.677	2.694
B2PLYPD3, gd3	def2tzvp	−	1.687	2.700
B2PLYPD3, gd3	def2tzvpp	−	1.694	2.703
wB97XD	def2svp	12.5	1.625	2.650
wB97XD	def2svp	29.3	1.620	2.647
wB97XD	def2svp	46.8	1.619	2.646
wB97XD	def2svp	108.9	1.617	2.645

**Table 3:** The external electric field at which the energy minima of the molecular (O-H···N) and ionic (O^−^···[H-N]^+^) forms of H-bonds in **2**–**6** are equal.

Method	2	3	4	5	6
Field, a.u. & PCM ε =	0.0004 12.5	0.0005 29.3	0.0015 29.3	0.0014 29.3	0.0027 29.3
Field, a.u. (gas-phase)	0.0044	0.0046	0.0075	0.0061	0.0082

**Table 4:** Gas-phase proton affinities of selected proton acceptors.

Acceptor	PA, kJ/mol	Acceptor	PA, kJ/mol	Acceptor	PA, kJ/mol
pyridine	936	2-nitrobenzoate	1382	Formate	1431
collidine	988	3,5-dichlorobenzoate	1379	Acetate	1447
benzoate	1421	4-nitrophenolate	1354	fluoride	1547
		F^−^···HCF_3_	1429		
